# Microbial contamination in herbal medicines: a serious health hazard to elderly consumers

**DOI:** 10.1186/s12906-019-2723-1

**Published:** 2020-01-23

**Authors:** Carolina Miranda de Sousa Lima, Mayara Amoras Teles Fujishima, Bruno de Paula Lima, Patrícia Carvalho Mastroianni, Francisco Fábio Oliveira de Sousa, Jocivânia Oliveira da Silva

**Affiliations:** 1 Course of Pharmacy, Laboratory of Toxicology, Department of Biological and Health Sciences, Federal University of Amapa, Juscelino Kubitschek Highway, KM-02, Jardim Marco Zero, Macapa, AP ZIP code: 68.903-419 Brazil; 2 Course of Medicine, Department of Biological and Health Sciences, Federal University of Amapa, Juscelino Kubitschek Highway, KM-02, Jardim Marco Zero, Macapa, AP ZIP code: 68.903-419 Brazil; 3 Faculty of Pharmaceutical Sciences, State University Paulista Julio Mesquita Filho, Araraquara, Rodovia Araraquara-Jaú KM 01, Machados, São Paulo, SP ZIP code: 14800-901 Brazil; 4 Course of Pharmacy, Laboratory of Quality Control and Bromatology, Department of Biological and Health Sciences, Federal University of Amapa, Juscelino Kubitschek Highway, KM-02, Jardim Marco Zero, Macapa, AP ZIP code: 68.903-419 Brazil

**Keywords:** Herbal medicines, Elderly individuals, Microbial contamination

## Abstract

**Introduction:**

The use of herbal medicine is on the rise worldwide, and safety issues associated with herbal medicines may have an exacerbated impact in elderly because this population has an increased susceptibility and sensitivity to health complications due to the aging process.

**Methods:**

This cross-sectional study was carried out at a primary health care unit in the city of Macapa, Brazil. The herbal medicines used and the sociodemographic characteristics of 123 voluntarily consenting participants were collected using a structured questionnaire. A total of 132 herbal medicines with oral or topical administration were donated by the elderly for microbial analysis before consumption, and 18 water samples used in the preparation of homemade herbal medicines were collected. Bacterial and fungal counts and identification of bacterial pathogens (*Escherichia coli*, Salmonella spp., *Pseudomonas aeruginosa* and *Staphylococcus aureus*) were performed according to the regulations of the Brazilian Pharmacopoeia and World Health Organization. Water analysis for the detection of coliforms and *E. coli* was carried out using Colilert® according to the manufacturer’s instructions and the techniques established by Standard Methods.

**Results:**

Of the study participants, 78.8% were women. Bacterial growth was observed in samples from 51.5% of study and 35.6% had fungal growth. A total of 31.8% of the herbal medicine samples exceeded the safety limits (CFU/g ≤ 10^5^), including 16.7% of the homemade herbal medicines and 15.1% of the commercial herbal medicines. It was also found that 31.0% of the samples exceeded the safety limit for fungal growth. The microorganisms most commonly isolated from the herbal medicines were *S. aureus* (49.2%), followed by Salmonella spp. (34.8%), *E. coli* (25.8%), and *P. aeruginosa* (14.4%). Of water samples analyzed, 77.8% were positive for total coliforms (1 ml) and in 66.7% water samples *E. coli* was detected (1 ml), making them unfit for consumption.

**Conclusions:**

The use of homemade and commercial herbal medicines is a major risk to the health of elderly who use these therapies due to the lack of microbial quality standards. We observed levels of viable bacteria and fungi that were above safety limits; in addition, we were able to isolate pathogenic bacteria from these herbal medicines.

## Background

It is estimated that approximately 80% of the population in developing countries uses traditional herbal medicines as part of their primary health care [1, 2]. This finding highlights the importance of research to support the development of traditional herbal medicine practices that provide appropriate, safe, and effective treatments [2–4]. Among the main safety risks related to herbal medicines is contamination by microorganisms of various kinds that may be adherent to leaves, stems, flowers, seeds, and roots from which herbal medicines are prepared. Alternatively, microorganisms can be introduced during harvesting, handling, open-air drying, preserving, and manufacturing [4–6].

Because of gradual devaluation of the knowledge associated with traditional health care-related practices [7], health surveys conducted in several countries have demonstrated the use of herbal medicines as a mainstream practice among elderly people compared with that among young adults [8]. According to the World Health Organization (WHO) [9], the definition of “elderly people” is established according to the socioeconomic level of each nation, with elderly individuals defined as being 60 years of age or greater in developing countries, while in developed countries, the age limit extends to 65 years.

The risk of microbial contamination may have an exacerbated impact in the elderly population because this population has increased susceptibility to the consumption of herbal medicines and sensitivity to health complications due to the aging process. The aging process, according to some studies [10–12], is characterized by morphological changes and physiological, biochemical and psychological factors that lead to a decrease in an individual’s ability to adapt to the environment.

Thus, it is important to consider the health care needs of this population. The prevalence of acute and chronic diseases increases with aging. This increase also leads to a considerable increase in the consumption of medications, and these factors predispose the geriatric population to the risks of polypharmacy as well as an increased occurrence of adverse events [13]. As a result, the presence of microbial contaminants in herbal products can adversely affect the health status of consumers due to their immunocompromised conditions and microbial infections, creating a health problem throughout the world. Therefore, the safety of consumers of herbal products is of the utmost importance [14], especially in elderly populations.

In the Brazilian Amazon, there is substantial consumption of herbal medicines due to cultural, social, and economic factors as well as the availability of a vast biodiversity of regional flora. However, there is no sufficient data that address the microbial quality of these herbal medicines. The present study evaluated the microbial quality (quantification of aerobic bacteria, identification of pathogenic bacteria and determination of presence of fungi) of herbal medicines (commercial and homemade) and of water used in the preparation of homemade herbal medicines consumed by the elderly population in the Brazilian Amazon (Macapa, Amapa State).

## Methods

### Study area, design and period

This cross-sectional study was carried out from May 1, 2016 to October 1, 2017 at the Frei Daniel Samarate primary health care unit in the city of Macapa, which is located in northern Brazil (latitude 00°02′18.84“N and longitude 51°03’59.10”W). The town has an estimated area of 6.503.458 km^2^, with a population of over 398.204, of which 20.508 are elderly individuals [15]. Macapa is situated on a small plateau in the Amazon in the southeast of the state of Amapa and has few land connections with other parts of Brazil.

### Source of population and study subjects

All types of herbal medicines used by the 123 voluntary participants were used as the study subjects. It was compulsory that these people were at least 60 years of age, nonindigenous (to establish ethical criteria because studies involving indigenous people and their knowledge/culture are required to follow specific ethical recommendations), and in perfect mental health (certified through the analysis of medical records). Sociodemographic characteristics were also collected using a structured questionnaire.

### Sample size and sampling technique

In the present study, a total of 132 (86 liquid, 25 semisolid and 13 solid) herbal medicines with an oral or topical administration were donated for microbial analysis by the 123 elderly participants before consumption. The samples were sold at different markets in the town of Macapa (commercial/industrialized or technically elaborated product) or prepared by the elderly individuals themselves (homemade/herbalists). A total of 18 water samples used in the preparation of herbal medicines by participants (homemade) were included as study subjects.

### Inclusion and exclusion criteria

Liquid, semisolid or solid preparations of herbal medicinal products administered orally or topically with or without further pharmaceutical processing stored in their original container (in the case of commercial products) or collected in sterile bottles provided by the researchers (in the case of homemade herbal medicines) were included in the study. Herbal medicines with other routes of administration and/or those that were delivered in unsuitable bottles (nonsterile or different from the original) were excluded from the study.

### Data collection and handling and transportation of specimens

Using aseptic techniques, 10 mL (liquid) or 10 g (semisolid or solid) of samples from each herbal preparation were collected using a sterile screw-capped bottle. Eighteen water samples used to prepare liquid herbal medicines were collected by immersing a 100-mL sterile screw-capped bottle in a home faucet of the elderly participants. All of the samples were transported to the Toxicology Laboratory of the Federal University of Amapa in a cold box within 1 hour of collection. Liquid specimens were refrigerated at 4 °C until processing.

## Bacterial and fungal counts

The microbial quality, including the isolation and identification of pathogenic bacteria from commercial and homemade herbal medicines, was tested according to the regulations of the Brazilian Pharmacopoeia [16] and WHO standards [3]. The tests were used to quantify the number of bacteria and fungi isolated that are able to grow aerobically in 1 g or 1 mL of sample.

The samples were homogenized by mixing vigorously with or without previous maceration, of 1-g (semisolid or solid samples, soluble in water - when the product was water soluble or tween - when the product was fat soluble - 1% sample solution) or 1-mL (liquid samples) quantities of samples were transferred to 9 mL of casein-peptone (KASVI®). Then, serial dilutions were made to achieve an appropriate concentration. All microbial analyses were carried out in triplicate. Briefly, serial dilutions were made, and viability was assessed using the pour plate method on tryptic soy agar (KASVI®) or Sabouraud dextrose agar (KASVI®) for bacterial counts and fungal identification, respectively. All dehydrated media were prepared according to the manufacturer’s instructions and seeded and incubated at 37 °C for 2–5 days for bacterial screening and at 25 °C for 5–7 days for fungal screening [3–16]. At the end of the incubation period, the number of colony-forming units per gram (CFU/g) was calculated by multiplying the average number of colonies by the dilution factor. The obtained CFU/g of sample was compared with WHO standards [3]. Samples that presented bacterial growth greater than 10^5^ CFU in 1 g of herbal medicine were considered unsatisfactory or inadequate according to WHO guidelines [3] for aerobic bacteria.

### Identification of Bacteria

For bacterial isolation and identification, the samples were diluted in water or Tween, according to the solubility, and homogenized by vigorously mixing. The 1-mL aliquots were transferred to 9 mL of tryptone soy broth (KASVI®) or lactose broth (KASVI®) and cultured at the recommended time and temperature [16]. All microbial analyses were carried out in triplicate. For investigating *Escherichia coli*, *Salmonella* spp., *Pseudomonas aeruginosa* and *Staphylococcus aureus*, MacConkey agar (KASVI®), cetrimide agar (KASVI®), EMB agar (KASVI®), mannitol salt agar (KASVI®), Brilliant Green agar (KASVI®), and triple sugar iron agar (KASVI®) culture media were used. At the end of the incubation period, pathogenic bacterial isolates were preliminarily characterized by colony morphology, Gram staining, and biochemical tests (oxidase, gas and catalase production).

### Water analysis

Detection of coliforms and *E. coli* in water samples was carried out in duplicate using Colilert® according to the manufacturer’s instructions and the techniques established by Standard Methods [17]. Yellow samples indicated the presence of coliform bacteria, and samples that were yellow and fluorescent when exposed to UV light (366 nm) indicated the presence of *E. coli*. Water samples were considered satisfactory or adequate, with an absence of total coliforms and/or *E. coli* in 100 mL, according to the Brazilian regulation for water quality for human consumption [18].

### Statistical analyses

BioEstat® 5.3 software was used with bilateral hypotheses (μ1 ≠ μ0), 95% CI (confidence interval) and α = 0.05 (*p* ≤ 0.05) for performing statistical analyses. The descriptive statistics of sociodemographic variables (mean and standard deviation) are presented. Analysis of variance (ANOVA) and *odds ratio* were also used.

## Results

Among the 132 herbal products analyzed, 83.3% were purchased from herbal shops or drugstores in different parts of the town of Macapa, while 16.7% were prepared from cultivated plants from a personal medicinal herb garden. Importantly, the samples include commercial/industrialized or technically elaborated product) or prepared by the elderly individuals themselves (homemade/herbalists). Of the study participants, 78.8% were women, with an average age of 69.4 ± 7.5 years. A total of 68.8% of the elderly participants were single, divorced or widowed, and 52.9% lived in suburban areas of the city. Approximately 23.1% of the participants were illiterate, and 50.5% had between 1 and 6 years of education. Regarding their monthly family income, 74% earned ≤588.80 dollars (Table 1).

**Table 1:** Demographic and socioeconomic characteristics of the elderly participants (*N* = 132)

Demographics and Socioeconomic Index	N	%
Gender		
Female	104	78.8
Male	28	21.2
Age group (years)		
60–69	78	59.1
70–79	39	29.5
≥80	15	11.4
Marital status		
Not married, widower or divorced	70	52.9
Married or in a stable union	62	47.1
Education level (years)		
No schooling	30	23.1
> 1≥6 years	67	50.5
≥7 years	35	26.4
Household income/month ($)^a^		
≤588.80	98	74.0
> 588.80≥2650.00	29	22.1
> 2.650.00	5	3.9

Macapa, Brazil, from 2016 to 2017

^a^US dollars based on the Brazilian central bank [19] on 01/08/2018 (R$3.24)

The liquid herbal medicines analyzed included teas, tinctures, potions, syrups, and oils, and the semisolids were gels, creams, and balms. The solids included macerated plant parts or powdered plants, both of which were intended to be mixed in water or food (Table 2). Among herbal medicinal products, 68% were composed of several plants (6 ± 1.6), while only 22% of the homemade herbal medicines were prepared with a single plant species.

**Table 2:** Uses and characteristics of homemade herbal medicines and herbal medicinal products sold in different markets of Macapa, Brazil, from 2016 to 2017

Uses and characteristics	Homemade herbal medicines N (%)	Commercial herbal medicine N (%)	Total N (%)
Oral			
Solid	3 (5.8)	8 (10.0)	11 (8.4)
Semisolid	5 (9.6)	0 (0)	5 (3.8)
Liquid	33 (63.5)	50 (62.5)	83 (62.9)
Topical			
Solid	4 (7.7)	0 (0)	4 (3.0)
Semisolid	2 (3.8)	14 (17.5)	16 (12.1)
Liquid	5 (9.6)	8 (10.0)	13 (9.8)
Total	52 (100)	80 (100)	132 (100)

Table 3 shows the ten most frequently reported herbal medicines used by elderly people along with their botanical names, reported properties and uses. *Lippia alba* (Mill.) N.E. Br (Cidreira) and *Peumus boldus* Molina (Boldo) were the most frequently consumed.

**Table 3:** Herbal medicines most frequently used by elderly people. Macapa, Brazil, from 2016 to 2017

Botanical name^a^	Popular name	Reported properties and uses	N (%)
*Lippia alba* (Mill.) N.E. Br	Cidreira	Relaxation and digestive problems	18 (13.6)
*Peumus boldus* Molina	Boldo	Digestive and liver problems	13 (9.9)
*Cymbopogon citratus* (DC.) Stapf	Capim-marino	Relaxation and digestive problems	11 (8.3)
*Carapa guianensis* Aubl.	Andiroba	Inflammation, bruises	8 (6.1)
*Copaifera langsdorffii* Desf.	Copaíba	Inflammation, infections	6 (4.5)
*Stryphnodendron adstringens* (Mart.) Coville	Babatimão	Infections, wound healing, pain, inflammation	6 (4.5)
*Costus spicatus* (Jacq.) Sw.	Canaficha	Kidney problems (diuretic effect)	3 (2.3)
*Arrabidaea chica* (Bonpl.) Verl.	Pariri	Pain, fever, inflammation and/or spasms	3 (2.3)
*Associations of plant species*		Sexual stimulants, inflammatory disease of the female reproductive system, rheumatic diseases, etc.	51 (38.6)
Others			13 (9.9)
Total			132 (100)

^a^The classification of botanical names was performed according to THE PLANTS LIST® database [20]. The botanical identification of the herbal medicines obtained in pharmacies was derived from the labels / packages, and the herbal medicines obtained in gardens, fairs and popular markets were identified by visual comparison with pictures and images from online herbariums (reportedly used by the interviewees to provide relief against illnesses)

For the evaluation of microbial contamination, the total number of viable bacterial and fungal colony-forming units per gram (CFU/g) were determined (Table 4). Among 132 samples of commercial and homemade herbal medicines, 51.5% had bacterial growth and 35.6% had observable fungal growth, independent of acceptable limits of contamination. A total of 31.8% of samples exceeded the safety limits (CFU/g ≤ 10^5^) according to WHO guidelines [3] for aerobic bacteria, including 16.7% of the homemade herbal medicines and 15.1% of the commercial herbal medicines. It was also found that 23.5% of samples exceeded the safety limits for fungal growth. The liquid herbal medicine preparations administered orally were the most frequently contaminated. No statistically significant difference (*p* = 0.6313 / ANOVA) was found between the bacterial CFU/g (quantitative tests) found in homemade herbal medicines and that found in commercial herbal medicines. There was also no statistically significant difference (*p* = 0.5277 / ANOVA) between the fungal and bacterial CFU/g detected in the analyzed samples. When the differences in contamination between pathogenic bacteria (qualitative test) were significantly different (*p* = 0.005 / chi-square), it was demonstrated that the homemade herbal medicines had a higher risk of contamination by pathogenic bacteria than the commercial herbal medicines.

**Table 4:** Determination of the total viable bacterial and fungal counts (CFU/g) in herbal medicine samples

Herbal medicine				Acceptable limits (CFU/g ≤ 10^5^)^a^ count/g or mL		Unacceptable limits (CFU/g > 10^5^)^a^ count/g or mL	
				Total viable aerobic bacterial count	Total viable fungal count	Total viable aerobic bacterial count	Total viable fungal count
Preparation	Use	Forms	Samples % (N)	% (N)	% (N)	% (N)	% (N)
Homemade herbal medicines	Oral	Solid	5.3 (7)	1.5 (2 ± 0.8)	1.5 (2 ± 1.1)	2.3 (3 ± 0.5)	3.0 (4 ± 1.8)
		Semisolid	1.5 (2)	0 (0 ± 0.2)	0 (0)	0.8 (1 ± 0.2)	0.8 (1 ± 1.0)
		Liquid	23.5 (31)	10.6 (14 ± 1.2)	3.0 (4 ± 1.1)	10.6 (14 ± 1.5)	3.0 (4 ± 2.3)
	Topical	Solid	3.0 (4)	0.8 (1 ± 0.3)	0 (0)	1.5 (2 ± 0.4)	1.5 (2 ± 2.0)
		Semisolid	2.3 (3)	0 (0)	0.8 (1 ± 0.2)	0 (0)	0.8 (1 ± 0.8)
		Liquid	3.8 (5)	0 (0)	1.5 (2 ± 1.0)	1.5 (2 ± 0.5)	1.5 (2 ± 1.4)
	Total		39.4 (52)	12.9 (17 ± 1.8)	6.8 (9 ± 0.9)	16.7 (22 ± 2.3)	10.6 (14 ± 1.9)
Commercial herbal medicines	Oral	Solid	7.6 (10)	0.8 (1 ± 0.5)	1.5 (2 ± 0.5)	3.0 (4 ± 0.5)	3.0 (4 ± 1.3)
		Semisolid	0 (0)	0 (0)	0 (0)	0 (0)	0 (0)
		Liquid	36.4 (48)	6.0 (8 ± 1.1)	3.0 (4 ± 1.2)	8.3 (11 ± 1.8)	8.3 (11 ± 2.3)
	Topical	Solid	0 (0)	0 (0)	0 (0)	0 (0)	0 (0)
		Semisolid	10.6 (14)	0 (0)	0 (0)	1.5 (2 ± 0.5)	1.5 (2 ± 1.0)
		Liquid	6.0 (8)	0 (0)	0.8 (1 ± 1.0)	2.3 (3 ± 0.8)	3.8 (5 ± 1.1)
	Total		60.6 (80)	6.8 (9 ± 1.1)	5.3 (7 ± 1.2)	15.1 (20 ± 1.5)	16.7 (22 ± 1.9)
Grand total			100 (132)	26 (19.7)	16 (12.1)	42 (31.8)	31 (23.5)

Macapa, Brazil, from 2016 to 2017

^a^Microbial contamination limits in herbal materials, preparations and finished products according to WHO standards [3]. All experiments were completed in triplicate, (N) represents absolute values as mean and standard deviation

The microorganisms most commonly isolated from the homemade herbal medicines and commercial herbal medicine (Table 5) were *S. aureus* (49.2%), followed by *Salmonella* spp. (34.8%), *E. coli* (25.8%), and *P. aeruginosa* (14.4%). The results from the microbiological analyses of the homemade herbal medicines showed that *S. aureus* was isolated from 88.5% of the samples, *Salmonella* spp. were isolated from 69.2% of the samples, *E. coli* was isolated from 53.8% of the samples and *P. aeruginosa* was isolated from 25.0% of the samples. Among the commercial herbal medicines analyzed, 23.8% contained *S. aureus*, 12.5% contained *Salmonella* spp., and 7.5% contained *E. coli* and *P. aeruginosa*. The odds of identifying *P. aeruginosa* (odds ratio = 0.208, *p* = 0.001, 95% CI 114 ≤ μ ≥ 0.377) and *E. coli* (odds ratio = 0.429, *p* = 0.002, 95% CI 0.255 ≤ μ ≥ 0.721) were low. We also did not find any association between the odds of identifying *S. aureus* (odds ratio = 1.200, *p* = 0.537, 95% CI 0.739 ≤ μ ≥ 1.947) and the odds of identifying *Salmonella* spp*.* (odds ratio = 0.661, *p* = 0.131, 95% CI 0.403 ≤ μ ≥ 1.086).

**Table 5:** Pathogenic bacterial species isolated from herbal medicines consumed by elderly individuals

Herbal medicine				Bacterial isolates^*^			
Preparation	Use	Forms	Samples % (N)	*Escherichia coli*^*a*^ % (N)	*Salmonella* spp*.*^*a*^ % (N)	*Pseudomonas aeruginosa*^*a*^ % (N)	*Staphylococcus aureus*^*b*^ % (N)
Homemade herbal medicines	Oral^a^	Solid	5.3 (7)	1.5 (2 ± 0.8)	1.5 (2 ± 0.2)	0 (0)	3.0 (4 ± 0.3)
		Semisolid	1.5 (2)	0 (0)	0 (0)	1.5 (2 ± 0.1)	0.8 (1 ± 0.2)
		Liquid	23.5 (31)	12.9 (17 ± 0.4)	15.9 (21 ± 0.6)	8.3 (11 ± 0.4)	20.4 (27 ± 0.9)
	Topical	Solid	3.0 (4)	3.0 (4 ± 0.6)	3.0 (4 ± 0.1)	0 (0)	1.5 (2 ± 0.2)
		Semisolid	2.3 (3)	2.3 (3 ± 0.4)	3.8 (5 ± 0.3)	0 (0)	6.8 (9 ± 0.5)
		Liquid	3.8 (5)	1.5 (2 ± 0.1)	3.0 (4 ± 0.1)	0 (0)	2.3 (3 ± 0.2)
	Total		39.4 (52)	39.4 (52)	69.2 (36)	25.0 (13)	88.5 (46)
Commercial herbal medicine	Oral^a^	Solid	7.6 (10)	0.8 (1 ± 0.2)	0 (0)	1.5 (2 ± 0.2)	6.1 (8 ± 0.6)
		Semisolid	0 (0)	0 (0)	0 (0)	0 (0)	0 (0)
		Liquid	36.4 (48)	1.5 (2 ± 0.3)	3.0 (4 ± 0.2)	1.5 (2 ± 0.1)	4.5 (6 ± 0.4)
	Topical	Solid	0 (0)	0 (0)	0 (0)	0 (0)	0 (0)
		Semisolid	10.6 (14)	0 (0)	3.0 (4 ± 0.1)	0 (0)	2.3 (3 ± 0.5)
		Liquid	6.0 (8)	2.3 (3 ± 0.4)	1.5 (2 ± 0.1)	1.5 (2 ± 0.1)	1.5 (2 ± 0.1)
	Total		60.6 (80)	60.6 (80)	12.5 (10)	7.5 (6)	23.8 (19)
Grand total			132 (100)	25.8 (34)	81.7 (46)	14.4 (19)	49.2 (65)

Macapa, Brazil, from 2016 to 2017

^*^Presence in 1 g or 1 mL of sample [3]. All experiments were completed in triplicate, (N) represents absolute values as mean and standard deviation

^a^Gram-negative, ^b^ gram-positive

Table 6 presents the results for the identification of total coliforms and occurrence of *E. coli* in water samples used to prepare homemade herbal medicines. Of 18 samples analyzed, 14 (77.8%) had total coliforms, and *E. coli* was observed in 12 (66.7%), making them unfit for human consumption

**Table 6:** Total coliforms and *E. coli* detection in different water samples used to prepare homemade herbal medicines (*N* = 18)

Parameter^a^	N	%
Total coliforms		
Yes	14	77.8
No	4	22.2
*E. coli*		
Yes	12	66.7
No	6	33.3

Macapa, Brazil, from 2016 to 2017

^a^Microbial contamination limits in herbal materials, preparations and finished products according to WHO standards [3]. All experiments were completed three times, and the results were reproducible

## Discussion

Herbal medicines are extensively used in Brazil due to the country’s diverse plant population, great sociodiversity, and conventional wisdom originating from three ethnic backgrounds (indigenous, African, and European) [21]. Presently, herbal medicines are used along with synthetic medicines to reduce health care costs for those individuals who have limited access to modern health care facilities [22] because these individuals do not have health insurance coverage and do not have much education [23]. Herbal medicines are inexpensive treatment options because they are easy to prepare or purchase in street markets that are common in the Amazon region.

Because of gradual devaluation of the knowledge associated with traditional health care-related practices [7], health surveys conducted in several countries have demonstrated greater use of herbal medicines as a mainstream practice among elderly people compared with that among young adults [8]. Regarding the sex of the elderly individuals studied, we observed that the majority of study participants were females, which can be justified by gender-based comparative studies on the knowledge of medicinal plants. These types of studies have demonstrated the social role of women (wives and daughters) in health care practices, including diagnosing illnesses, knowing prognoses, and being responsible for implementing the first treatments [2–6]. In a study of medicinal plants used by the local people of the Tafila area of Jordan, the traditional uses of these plants were investigated, and women were identified as the sole source of knowledge on herb use [24].

Among the herbal medicines most frequently used by the elderly population of the Brazilian Amazon, *Lippia alba* (Mill.) N.E. Br (Cidreira) and *Peumus boldus Molina* (Boldo) were the most common. These herbal medicines are consumed not only because of their therapeutic properties for the gastrointestinal tract [7–25] but also because of their calming and relaxing effects, which makes them multifunctional herbs. Generally, herbal medicines are used for treating simple diseases, such as digestive or respiratory diseases and general pain, whereas synthetic medicines are primarily used for blood pressure problems, general pain, and endocrine and nutritional diseases [26]. In studies that have included the Brazilian Atlantic Forest population, authors reported some species of medicinal plants that are commonly used in these regions, such as *L. alba* (Mill.) N.E. Br, *Vernonia sp*., *C. citratus, P. boldus Molina, Matricaria chamomilla L,* and *Zingiber officinale* [26].

The integration of plant-based medications into the primary health care system of developing countries is being expanded; however, safety issues continue to be neglected [1–3]. Bacterial and fungal contamination are frequent, especially in homemade herbal medicines, with CFU/g levels above recommended national [16] and international [3] standards. Herbal medicines in liquid pharmaceutical form for oral use presented the highest microbial contamination, and they were also the most consumed products among elderly people. Presumably, the proliferation of microorganisms may result from the failure to control moisture levels of herbal medicines during transportation and storage, as well as from the failure to control the temperatures of liquid forms and finished herbal products [3]. Moreover, most medicinal plants are prepared in an open environment in nonhygienic conditions that gradually lead to contamination with enteric pathogens with public health importance [26]. In the present study, of the 132 herbal medicine preparations, 31.8% were above the limit of acceptable bacterial counts according to WHO standards [3], indicating a risk in the consumption of the analyzed products. In other studies, bacterial counts were measured in herbal materials and herbal medicines [4–6, 14, 27–29], indicating risks in the consumption of these products.

Microbial analyses detected the presence of *E. coli, Salmonella* spp*.* and *P. aeruginosa*, which are all indications of fecal contamination, revealing poor hygiene conditions in the preparation and storage of these herbal medicines. The presence of bile-tolerant gram-negative bacteria belonging to the family *Enterobacteriaceae* is an important indicator of hygienic precariousness, inadequate processing or postprocessing contamination. Pathogens of this family are potential causative agents of foodborne diseases, increasing the risk for consumers to develop intestinal tract infections [30]. Since the detection of *E. coli* is indicative of fecal contamination, it can be concluded that the herbal medicines that contained this microorganism were contaminated directly or indirectly by human or animal feces and were therefore unsuitable for consumption [31].

The analyses of this study also detected the presence of *S. aureus*, which can cause staphylococcal gastroenteritis, scalded-skin syndrome, and folliculitis, among other diseases [31]. A study that also evaluated the microbial quality of herbal medicines showed similar results, with 47.6% of samples contaminated with *E. coli*, 33% of samples contaminated with *Salmonella* spp., and 71.4% of samples contaminated with *S. aureus* [32]. Pathogenic bacteria, such as *E. coli, Salmonella* spp*., Shigella, S. aureus* and *P. aeruginosa*, were also found in other studies [30, 31]. These contaminations were probably caused by unsafe collection, transportation, drying, preparation, storage or dispensing processes of the herbal medicines [30].

The quality of the water used in the preparation of herbal medicines may have contributed to the high level of bacterial contamination observed for the homemade herbal medicines. Drinking water should be free from pathogenic microorganisms and bacteria that indicate fecal contamination [33]. However, 66.7% of the water samples used for the preparation of herbal medicines were not adequate for consumption. The problem may be related to the lack of basic sanitation and the inadequate management of human and animal dejections incorporated into the soil, which are the most important factors in the contamination of water resources [34]. The water quality and notions of hygiene are likely related to the differences in the contamination of pathogenic bacteria in the herbal medicines and herbal medicine products identified in this study.

Characterization of fungal colonies, although not required by the Brazilian Pharmacopoeia [16], is important in assessing the risk of analyzed products. Microscopic analysis suggested fungal contamination that was similar to another study that demonstrated the presence of fungal species known to be able to produce mycotoxins, such as *Aspergillus niger, Aspergillus ochraceus, Aspergillus flavus* and *Aspergillus parasiticus* [35]. These species may pose a risk when present in products used orally. Many studies have also demonstrated the presence of mycotoxins in preparations derived from medicinal plants [34]. The severity of mycotoxins depends on the toxicity, degree of exposure, age and nutritional status of the individual and the possible synergistic effects of other chemical agents to which they are exposed [36].

Good manufacturing practices in pharmacies or industries that handle herbal medicines are essential. Beyond compliance, following current national and international legislations, such as European Scientific Cooperative on Phytotherapy and Brazilian Pharmacopoeia, is important [3, 16–38]. It is also essential to monitor establishments that market herbal medicines by checking that they have a license from the health authority for this trade and that the products are registered and authorized for consumption [39], as cases of falsification or commercialization of irregular herbal medicines may occur.

In search of solutions for the irrational use of herbal medicines, programs were created to provide standards for the correct use of medicinal plants and select them according to their efficacy and safety, replacing the routine empirical use carried out by communities [40–42]. These initiatives, however, were not sufficient to guarantee the triad of efficacy, safety, and quality of the products used in herbal medicine, resulting in the marketing and consumption of products that do not meet the minimum criteria of microbial quality standards [3]. Other countries that culturally use herbal medicines [5, 7, 14, 22, 27, 41, 42] have also faced issues of quality incompliance in herbal medicines because of the presence of pathogenic bacteria. This dynamic shows that quality standards and product safety criteria have not yet been achieved in this complementary medical practice, which probably results in reduced teaching on herbal medicines in medical schools.

Pharmacovigilance of medicinal plants is also important and requires the collection of more information on the methods of preparation and administration, adverse events, contraindications and precautions for a better risk-effectiveness ratio [43]. The need for more effective monitoring of the commercialization and consumption of herbal medicines by elderly individuals has become evident because the risks are real and significant. Strategies for health education, campaigns and workshops for elderly people on the safe use of these therapies are essential for public health and patient safety.

## Conclusions

This study demonstrated the presence of aerobic bacteria and fungi above the acceptable limits as well as the presence of pathogenic bacteria in samples of herbal medicines used by elderly individuals in the Brazilian Amazon region. These findings demonstrate important risks for elderly individuals associated with the use of herbal medicines and the need for surveillance and the establishment of stricter control procedures in the production/preparation and marketing of these herbal medicines to guarantee quality. These steps will help to avoid additional risks to the elderly population of the city of Macapa in the consumption of herbal medicines, which is a common cultural health care habit in this Brazilian region. Control programs for the sale of these herbal medicines should be implemented by national regulatory agencies to prevent or reduce the consumption of products outside the minimum standards of quality. In addition, campaigns linked to primary health care units or family health programs with the aim of guiding the proper preparation of herbal medicines (avoiding microbial contamination) should be initiated in the region.

## Data Availability

All available data can be obtained from the corresponding author. All data will be shared in a way that safeguards the confidentiality and anonymity of the study participants.

## Abbreviations

CFU/g: Forming Units Colonies for 1 g
CI: (Confidence Interval)
WHO: World Health Organization
